# Schwann cell remyelination of the central nervous system: why does it happen and what are the benefits?

**DOI:** 10.1098/rsob.200352

**Published:** 2021-01-27

**Authors:** Civia Z. Chen, Björn Neumann, Sarah Förster, Robin J. M. Franklin

**Affiliations:** Wellcome-Medical Research Council Cambridge Stem Cell Institute, University of Cambridge, Cambridge Biomedical Campus, Cambridge CB2 0AH, UK

**Keywords:** Schwann cells, remyelination, central nervous system, oligodendrocyte progenitor cells, astrocytes, myelin

## Abstract

Myelin sheaths, by supporting axonal integrity and allowing rapid saltatory impulse conduction, are of fundamental importance for neuronal function. In response to demyelinating injuries in the central nervous system (CNS), oligodendrocyte progenitor cells (OPCs) migrate to the lesion area, proliferate and differentiate into new oligodendrocytes that make new myelin sheaths. This process is termed remyelination. Under specific conditions, demyelinated axons in the CNS can also be remyelinated by Schwann cells (SCs), the myelinating cell of the peripheral nervous system. OPCs can be a major source of these CNS-resident SCs—a surprising finding given the distinct embryonic origins, and physiological compartmentalization of the peripheral and central nervous system. Although the mechanisms and cues governing OPC-to-SC differentiation remain largely undiscovered, it might nevertheless be an attractive target for promoting endogenous remyelination. This article will (i) review current knowledge on the origins of SCs in the CNS, with a particular focus on OPC to SC differentiation, (ii) discuss the necessary criteria for SC myelination in the CNS and (iii) highlight the potential of using SCs for myelin regeneration in the CNS.

## 1. Overview

Demyelinating diseases comprise a diverse spectrum of disorders including the autoimmune disease multiple sclerosis (MS) in the central nervous system (CNS), Guillain Barre syndrome in the peripheral nervous system (PNS), and genetic disorders such as leukodystrophies (CNS) and Charcot–Marie–Tooth disease (PNS). Direct damage to the myelin sheath or to the myelinating cells, oligodendrocytes in the CNS and Schwann cells (SCs) in the PNS, can arise in consequence to genetic mutations, trauma, metabolic deficiencies or exposure to inflammation or toxins [1]. In both the CNS and PNS, the loss of myelin sheaths from otherwise intact axons, termed demyelination, is followed by a spontaneous, regenerative response, called remyelination. In the PNS, this process is mediated by surviving SCs, whereas in the CNS, this can be performed either by surviving oligodendrocytes or oligodendrocytes newly generated from oligodendrocyte progenitor cells (OPCs), a population of adult multipotent progenitors that are widespread throughout the CNS [2]. In this latter, better-understood form of CNS remyelination, OPCs migrate to the lesion site, proliferate and predominantly differentiate into new myelin-forming oligodendrocytes. However, SCs, the myelinating glia of the PNS, can also contribute to this CNS regenerative response [3,4]. Remyelination of central axons reverses conduction deficits and protects axons from secondary degeneration [5]; however, whether oligodendrocyte- or SC-mediated remyelination can achieve this to equal degrees is not known. Although the CNS has historically been seen as an organ with poor regenerative capacity due to the limited ability to regenerate neurons, the glial compartment is endowed with a remarkable regenerative capacity. Thus, while the need for remyelination therapies remains unmet, developing methods to harness the remarkable regenerative potential of myelinating glia represents an exciting avenue of research.

## 2. Schwann cells in the central nervous system

In this review, we focus on the intriguing observation that SCs, the myelinating cells of the PNS, can also be detected in the CNS of multiple sclerosis (MS) [4,6,7], neuromyelitis optica [8] and in spinal cord injury patients [9–11]. SC involvement in CNS remyelination is also a feature of a variety of demyelinating disease models, including experimental autoimmune encephalomyelitis (EAE) [12–14], focal compressive or contusive lesions and after local injections of toxins such as lysolecithin, 6-aminonicotinamde and ethidium bromide [3,15–17]. For many years, the predominant hypothesis regarding the origin of CNS-SCs argued that they invaded demyelinated regions of the CNS from the PNS following the breakdown of the *glia limitans* (for review see [18]). The observation that they tend to occur close to peripheral nerve roots is consistent with this explanation [19,20]. However, findings from genetic lineage tracing studies provided irrefutable evidence that the majority of CNS-SCs are derived from OPCs [21,22].

That CNS progenitors can give rise to SCs is surprising since ontogenetically the CNS and PNS diverge from one another at the early developmental stage of gastrulation when the neural tube is formed: oligodendrocytes derive from the neuroepithelium [23,24] while SCs originate from the neural crest [25,26]. A distinction between CNS and PNS myelinating glia can be further traced phylogenetically back to when myelin first appears in evolution at the divergence between the cartilaginous and bony fish [27]. Thus, the presence of SCs in the CNS is an unexpected phenomenon requiring SCs to overcome a developmentally determined spatial segregation, to reconcile evolutionary divergence and to exist in an environment defined by CNS glia. Although many open questions regarding the mechanisms and long-term consequences of SC-mediated CNS remyelination remain, evidence from SC transplantation studies demonstrate their promising repair potential [28–30]. It is thus hoped that enhancing SC remyelination may be a powerful strategy to complement oligodendrocyte remyelination and to attenuate axonal loss in the CNS. In this article, we first describe the developmental and functional differences between oligodendrocytes and SCs and then review current knowledge on the central origins of SCs. The potential mechanisms of OPC to SC differentiation will be examined, before finally discussing the therapeutic potential of SC-mediated remyelination as a treatment for CNS demyelinating diseases.

## 3. Developmental differences between oligodendrocytes and Schwann cells

**Figure 1.**
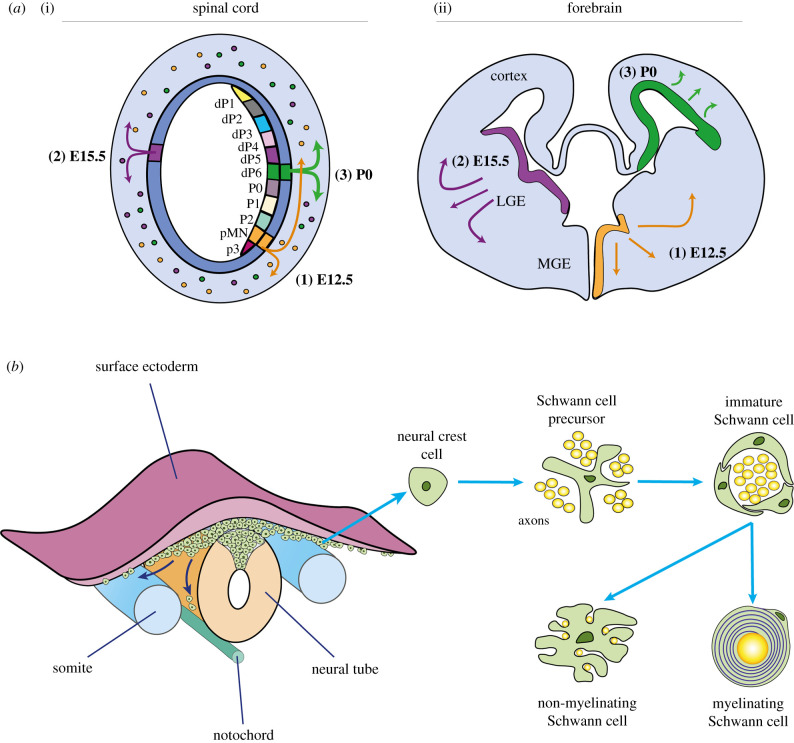
Origins, migration and development of OPCs and Schwann cells. (*a*) In the rodent spinal cord, OPCs originate from the pMN domain in the ventral ventricular zone at approximately embryonic (E)12.5. This is followed by a second wave of progenitors in more dorsal regions, which then migrate throughout the spinal cord to myelinate white matter tracts [23]. Similarly, in the rodent telencephalon, the first wave of OPCs arise from ventral progenitor cells in the medial ganglionic eminence (MGE) at E12.5. Subsequently, a second wave of OPCs is generated several days later at about E15.5 by dorsal progenitor cells in the lateral ganglion eminence (LGE). After birth, cortex-derived progenitors give rise to the final wave of OPCs. (*b*) During the formation of the neural tube, neural crest cells arise from the tips of the neural folds. Neural cells initially form and accumulate at the dorsal surface of the tube, but soon migrate along with different pathways to differentiate into SC precursor cells, as well as progenitors of melanocytes, autonomic neurons, dorsal root sensory glia, chromaffin cells and other peripheral glia). SC precursor cells then transition into immature SCs upon neuregulin-1 (NRG1), fibroblast growth factor 2 (FGF2) and Notch signalling. While all immature SCs are thought to possess myelinating potential, only immature SCs in close association with large-diameter axons differentiate into myelinating SCs. By contrast, SCs that associate with smaller-diameter axons form bundles of non-myelinating Remak cells [32].

Oligodendrocytes, the myelinating glia of the CNS, derive from OPCs which, for the embryonic spinal cord, mostly originate from restricted regions within the motor neural progenitor domain (pMN) of the ventral germinal zones (VZ). Ventrally derived OPCs then migrate dorsally and laterally to populate the entire white matter of the spinal cord, while the remaining approximately 20% of OPCs arise from progenitors of the dorsal ventricular zone in a later wave of OPC production to myelinate the dorsal spinal tracts [31]. An analogous event occurs in the developing brain, where OPCs first appear in the ventral VZ of the medial ganglionic eminence (MGE) and are followed by the production of dorsal OPCs in the lateral ganglion eminence (LGE). These newly generated OPCs migrate laterally and dorsally away from the MGE and LGE to spread throughout the developing cerebral cortex. After birth, a final wave of cortically derived OPCs settle within the cortex, while the first wave of MGE derived OPCs are eventually eliminated from the cortex through unknown mechanisms (figure 1*a*) [23,24]. Thus, in the adult mouse, OPCs in the brain are predominantly cortically derived, with an approximately 20% contribution from the LGE [33]. From these diverse origins, OPCs remain proliferative while migrating laterally and dorsally towards their appropriate destinations within the developing nervous system. Throughout this process, OPCs are reliant upon extracellular cues including mitogens such as platelet-derived growth factors (PDGFs) and fibroblast growth factors (FGFs), as well as contact-mediated signalling via ECM components for directional guidance [34]. Once in position, promoters of terminal differentiation, including thyroid hormones and insulin-like growth factor-1 (IGF-1), converge upon Olig2 to synergistically drive Sox10-mediated activation of Myrf (myelin regulating factor) [35–38]. In turn, Myrf initiates core programs to drive oligodendrocyte differentiation and myelin gene expression, to allow myelination to proceed [39,40].

SCs, in contrast, derive from multipotent, neural crest cells that detach from the dorsal neural tube and migrate over large distances to generate neurons and glia of the PNS, as well as other cell types including myofibroblasts, chondrocytes and melanocytes [41–43]. Migrating neural crest cells transition into SC progenitors, before differentiating into immature SCs (figure 1*b*). Although all immature SCs are thought to possess the capacity to upregulate myelinogenic programs, these cells can only terminally differentiate into mature myelinating cells when they associate with axons greater than 1 μm in diameter through a process known as radial sorting [32,44]. By contrast, SCs that associate with smaller-diameter axons form bundles of non-myelinating Remak cells [32,45]. Prior to complete maturation, SC survival and dynamics are strongly influenced by the molecular interactions between the SC and the axon [41,46], of which the neuronally derived factor, neuregulin-1 (NRG1), is particularly critical for promoting SC survival and for stimulating myelination [47]. Subsequent terminal differentiation is tightly regulated by a transcription factor network involving Sox10, Oct6 and Krox20 [48–50].

## 4. Functional differences between oligodendrocytes and Schwann cells

The vertebrate nervous system is equipped to execute complex motor, sensory and cognitive functions due, in part, to the evolutionary emergence of myelin. Myelination involves the wrapping of a multi-layered-lipid membrane around axons by glial cells–oligodendrocytes in the CNS, and SCs in the PNS. By insulating the axonal segments, myelin enables saltatory conduction [51,52], which accelerates action potential propagation up to 100-fold compared to nonmyelinated axons of the same diameter. Importantly, myelin also provides essential trophic support to maintain normal axonal transport and to ensure long-term axon survival [53]. The biogenesis of myelin requires temporal and spatial coordination of intricate machinery, whereby glial cells need to select axon targets for myelination and to construct myelin sheaths of the correct dimension and protein composition. In the PNS, SCs invade developing nerve bundles and subsequently associate with large-diameter axons in a 1 : 1 relationship. Once destined for myelination, multiple SCs line up along the axonal tracts to each extend their plasma membrane around axons in a spiral fashion, forming internodes with intervals, termed the Nodes of Ranvier, in between [54]. In the CNS, newly differentiated oligodendrocytes extend filopodia-like processes into the surrounding environment, where interactions between axonal and oligodendrocyte cell-adhesion molecules help to stabilize or to retract preliminary myelin sheaths [55]. Since oligodendrocytes can each myelinate multiple axons, it is thought that their dynamic processes may serve to regulate oligodendrocyte density and to ensure evenly spaced nodes [54]. Thus, while SCs and oligodendrocytes differ in their process to establish contact with axons, it is remarkable that oligodendrocytes and SCs both independently produce myelin that are remarkably similar but far from identical for the same purposes.

**Figure 2.**
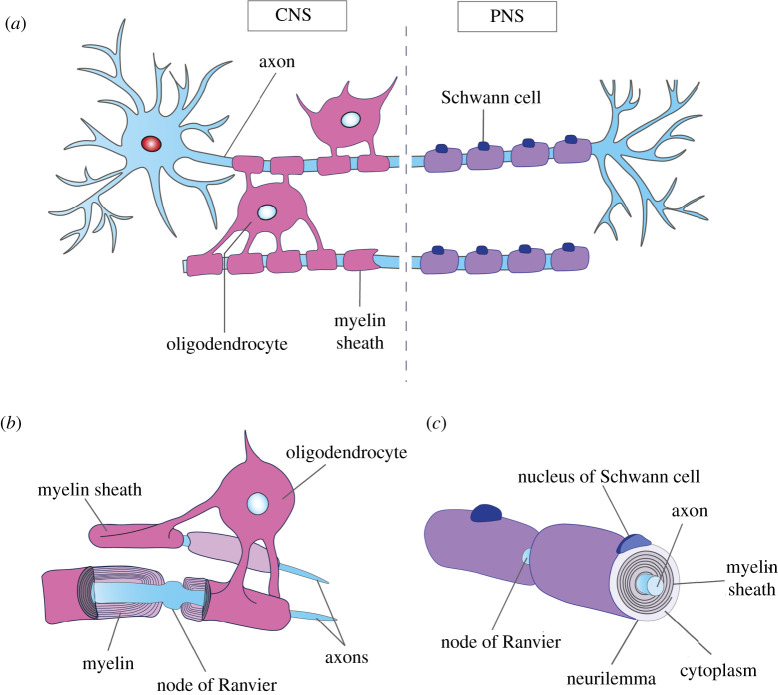
Comparison of central and peripheral myelination. (*a*) Each oligodendrocyte in the CNS can extend cytoplasmic projections to form multiple, multi-layered myelin sheaths (pink) around different axons, whereas each SC in the PNS completely wraps around a single axon by laying down multiple layers of cell membrane, of which the innermost layers constitute the myelin sheath (purple). (*b*) There is typically one oligodendrocyte between two nodes of Ranvier, which is not covered by plasma membrane to allow action potentials to jump from node to node. (*c*) Compared to oligodendrocytes, SC myelinated axons possess thicker myelin sheaths. SCs also have an enlarged non-axonal domain due to the extra presence of the cytoplasm and nuclei, which is covered by the outermost layer, called the neurilemma.

Even though the overall morphology of myelin is fairly well conserved, central and peripheral myelin are characterized by distinctive features (figure 2). In the PNS, SCs closely associate with the axon and each myelinate a single internode, while oligodendrocytes elaborate myelin sheaths around up to 60 different axons. Reminiscent of the cruder forms of myelin in non-vertebrate phyla, SCs only myelinate peripheral axons greater than 1 μm in diameter, whereas smaller axons are grouped into Remak bundles and loosely enveloped, but never myelinated [32]. Oligodendrocytes, on the other hand, do not perform radial sorting, or require axonally derived NRG1 signals to myelinate axonal segments [56]. Instead, the CNS appears to have evolved additional regulatory mechanisms involving other growth factors and signalling systems, particularly since oligodendrocytes can myelinate synthetic nanofibres independently of axonal signals [57]. Furthermore, while oligodendrocytes tend to similarly myelinate larger-diameter axons, in areas such as the optic nerve or the cortex they can also myelinate small, 0.2 μm-diameter axons [58,59]. Once myelinated, PNS axons are ensheathed in thicker myelin sheaths compared to CNS axons of the same diameter and show greater periodicity of spiral wrapping, probably due to differences in protein composition (table 1) [58].

**Table 1.** Relative abundance of major myelin proteins by mass spectrometric quantification.

myelin protein	CNS myelin (%) [60]	PNS myelin (%) [61]	associated disease	effect on myelination
proteolipid protein (Plp1)	17	0.2	Pelizaeus–Merzbacher disease; spastic paraplegia type 2	hypomyelinating leukodystrophy
myelin basic protein (Mbp)	8	8	18q deletion syndrome	dysmyelination
myelin protein zero (P0)	ND	21	Charcot–Marie–Tooth neuropathy	hypomyelinating neuropathy
periaxin (Prx)	ND	16	Charcot–Marie–Tooth neuropathy	hypomyelinating neuropathy
cyclic nucleotide phosphodiesterase (Cnp)	4	0.5	catatonia-depression syndrome upon aging	white matter neuroinflammation
myelin-associated glycoprotein (Mag)	1	0.3	autoantibody-mediated neuropathy	—
myelin oligodendrocyte glycoprotein (Mog)	1	ND	narcolepsy 7	—
sirtuin 2 (Sirt2)	1	ND	NA	NA
Claudin 11 (Cldn11)	1	ND	NA	NA
fatty acid synthase (Fasn)	1	ND	NA	NA
Band 4.1-like protein G (Epb4.1l2)	ND	1	NA	NA
others	67	52	—	—

Based on available fossil evidence, it is hypothesized that myelin was first acquired in restricted regions of the primitive nervous system to facilitate escape from predators. Subsequently, the increase in the size of vertebrates provided a strong incentive for widespread acquisition of myelination programs to enable faster impulse transmission across the longer nerves [62]. A common origin for myelination, rather than separate *de novo* development in each part of the nervous system, is suggested by the overlap in common myelin proteins (MBP, PLP and MAG). Taken together, evolution has produced two distinct glial cells that share many of the same features, as well as the same task of myelinating axons in the nervous system. The unique features of central and peripheral myelin allow each myelinating cell to better facilitate the neural and trophic demands of the CNS and PNS. These differences hold important therapeutic implications for the use of SCs for CNS remyelination. Thus, while SCs have been observed to spontaneously remyelinate central axons, whether SCs can fully substitute for the myelination provided by oligodendrocytes is unclear.

## 5. Sources of central nervous system-resident Schwann cells

The origin of CNS-resident SCs has been a matter of some debate. It was initially hypothesized that peripheral SCs invaded demyelinated regions of the CNS following the breakdown of the *glia limitans*, the structure made by astrocytic end feet that provides the ‘skin’ of the CNS [18]. This view is based on early observations that SCs in the CNS appear in regions with proximity to cranial or peripheral nerve roots, including the spinal cord, brain stem and cerebellum [19,20]. More recently, genetic fate-mapping studies have confirmed that a subset of Foxj1 expressing Remak cells can be recruited to remyelinate the CNS [63].

However, experiments showing that purified CNS-derived cell preparations, when transplanted into demyelinated CNS lesions, generated both oligodendrocytes and SCs suggested that some CNS-SCs might also be of CNS origin [64–67]. Since the purity of the transplanted preparations could never be proven to be absolute, it required the advent of genetic lineage tracing studies, using reporter proteins that were expressed exclusively in adult OPCs, to irrefutably demonstrate that CNS-resident SCs can be derived from OPCs [21,22,68]. Similar to peripheral SCs, OPC-derived SCs express proteins such as periaxin and P0, which are unique to PNS myelin [69,70], form a 1 : 1 association with axons and show the clear organization of internodes [21]. Together, these studies demonstrated that adult OPCs possess a wide differentiation potential, which, when challenged with damage-induced changes in the microenvironment, allow their differentiation into SCs of neural crest lineage.

## 6. Mechanisms of oligodendrocyte progenitor cells to Schwann cell differentiation

**Figure 3.**
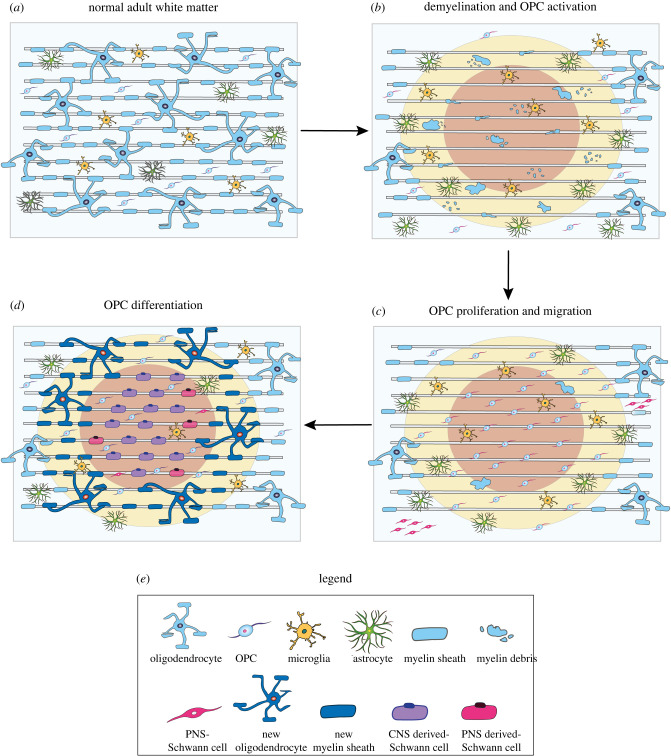
Schematic of Schwann cell remyelination. (*a*) Under homeostatic conditions, astrocytes, microglia, oligodendrocyte progenitor cells (OPCs) and myelinating oligodendrocytes are dispersed throughout the normal adult white matter. SCs are absent from the CNS. (*b*) Following demyelination oligodendrocytes and myelin are lost. In some instances, astrocytes can also be damaged. Subsequently, OPCs in in the vicinity of the lesion area are activated. (*c*) Activated OPCs are recruited into the lesion area by the release of pro-migratory and mitogenic factors, and the demyelinated region is repopulated by new OPCs. At the same time, a small subset of peripheral SCs transgress into the CNS due to breaks in the *glia limitans*. (*d*) Typically, the lesion centre (red) contains the fewest number of surviving astrocytes, and in the absence of astrocytic inhibition, OPCs differentiate into SCs, which form a 1 : 1 association with the axon, resulting in a single myelin sheath. Conversely, oligodendrocyte differentiation and remyelination predominates at the lesion border (yellow), where astrocytes are present. In a minority of cases, peripherally derived SCs migrate into the lesion site, where they make contact and myelinate exposed axons. Remyelination is mostly complete within 3 weeks after lesion.

How do OPCs activate a differentiation programme to produce cells of neural crest descent? Observations from both transplantation studies and spontaneous remyelination indicate that SC remyelination generally occurs in areas that are devoid of astrocytes [17,71], suggesting that the OPC to SC fate is gated by astrocytes (figure 3). In support of astrocyte inhibition, transplanted SCs show poor survival and minimal migration in lesions containing reactive astrocytes [71,72]. Similarly, few OPCs differentiate into SCs when co-transplanted with astrocytes into CNS lesions [18,73]. By contrast, endogenous and exogeneous SCs can widely remyelinate axons when astrocytes are experimentally ablated [74] or when their activation is blocked [75]. Subsequently, analysis of the lesion environment revealed significant upregulation in BMP, specifically BMP4 transcripts, suggesting that BMP signalling might be instructive for SC fate acquisition. Consistent with this finding, SC remyelination was not observed, even within astrocyte-free lesion areas, when BMP signalling was inhibited with Noggin [73]. Taken together, these studies proposed that OPCs differentiate into SCs in the presence of high BMP signalling, but only if these signals are not opposed by astrocytes in the lesion environment.

**Figure 4.**
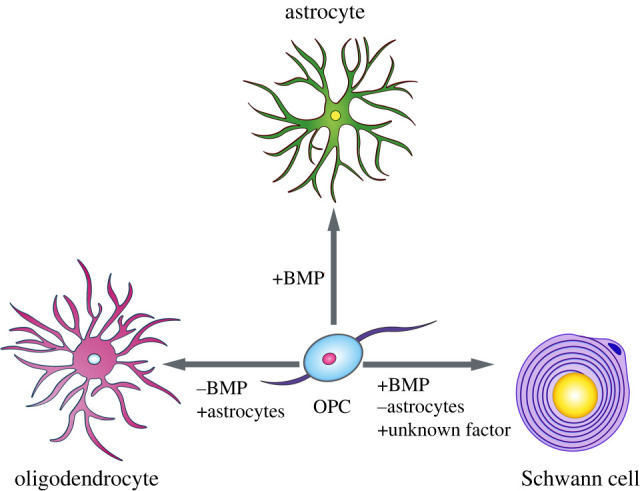
OPC cell fate decisions OPCs are multipotent and can differentiate into oligodendrocytes, astrocytes or SCs. OPC cell fate decisions are in part, dependent upon BMP signalling. When BMP signalling is blocked, such as when OPCs are in proximity to BMP suppressive astrocytes, OPCs predominantly differentiate into oligodendrocytes *in vivo* and *in vitro.* Conversely, when OPCs are exposed to high BMP signalling in culture, OPCs readily form astrocytes [76,77]; however, *in vivo,* few OPCs have been observed to produce astrocytes. Lineage tracing studies have demonstrated that following demyelinating injuries, adult OPCs can differentiate into SCs in astrocyte deficient areas [21]. Although BMP signalling is high within the lesion environment, BMP alone is not sufficient to produce OPC-derived SCs *in vitro*.

However, when pure OPCs were cultured in the absence of astrocytes or BMP signalling, OPCs only differentiate into astrocytes [76,77], suggesting that the BMP hypothesis does not fully capture the conditions needed for OPC to SC fate commitment (figure 4). Recently, transcriptomic profiling found increased BMP4 and also Wnt signalling in the lesion microenvironment due to a loss of reactive astrocytes that secrete the competitive BMP/Wnt antagonist, Sostdc1 [78]. From these findings, we would expect that unopposed BMP and Wnt signalling will instruct OPCs to differentiate into SCs: however, unpublished work from our laboratory found no significant increase in SC-remyelinated axons following demyelination in Sostdc1 KO mice. NRG1, which signals through the ErbB tyrosine kinase receptors, has also been identified as a molecular signal that allows OPCs to form SCs [79]. Although the authors showed that ablating ErbB3/4 decreases the generation of Schwann cells from endogenous central progenitors, it is yet to be determined whether increasing NRG1 activity in OPCs is sufficient to induce SC differentiation. Therefore, while BMP/Wnt and NRG1/ErbB signalling appear to help prime OPCs towards a SC fate, it is still unclear what key mediators are necessary to drive this alternative OPC cell fate decision. The close association of remyelinating SCs with blood vessels [80] makes it intriguing to speculate that the yet unidentified factor is blood derived or provided by the niche created by endothelium or the cells associated with the vasculature.

## 7. Therapeutic applications of Schwann cells in the central nervous system

Oligodendrocyte lineage cells, neural progenitor cells and olfactory ensheathing cells, among other cell types, have been shown to remyelinate CNS axons when transplanted directly into experimentally induced areas of demyelination [81,82]. However, as an intrinsic myelinating glia cell of the PNS, SCs are equipped to aid nerve impulse transmission and to provide neurotrophic support by generating *bona fide* myelin sheaths around exposed axons. These new nodes formed by remyelinating SCs, exhibit a mature configuration of voltage-gated sodium ion channels [83] and can be stably maintained for more than one year in rodents [84]. Transplanted SCs can myelinate central axons, which improves or restores axonal conduction velocity in demyelinated axons [29,85]. Even when oligodendrocyte remyelination is inhibited [86], SC remyelination in the CNS correlates with improved locomotion and neurological function [87,88]. In addition to restoring myelin sheaths, SC myelin tends to be spared from autoimmune attacks affecting oligodendrocytes or central myelin in diseases such as multiple sclerosis, presumably due to differences in protein expression between central and peripheral myelin (table 1) [86,87]. It is thus postulated that SC-remyelinated axons could be more resistant to CNS demyelinating diseases. Taken together, the regenerative and neuroprotective capacity of SCs make them attractive candidates for remyelinating therapies.

In addition to remyelinating central axons, there has been much interest in the use of SCs to aid regeneration of damaged CNS axons. Given the contribution of the regenerative SC in peripheral nerve damage [89], it was hoped that transplanting SCs into the CNS can similarly promote axonal regrowth. Indeed, early studies demonstrated that transplanting peripheral nerve grafts into the transected rat spinal cord was sufficient to help promote regeneration of the injured host axons [90]. Similarly, transplanted SCs, derived from purified cultures of SCs, taken from the peripheral nerve of rodents and humans, can remove myelin debris and release growth factors to make the lesion environment more permissive for axonal regrowth [91,92]. Moreover, SCs provide a scaffold to guide regenerating axons towards their intended target site and can remyelinate newly regenerated axons [89]. Together, these studies showed that SCs modify the lesion environment to facilitate central axon regeneration. To date, the therapeutic use of autologous SCs for spinal cord injury has passed phase 1 clinical trials [93,94]. Despite the excellent regenerative response by SCs, in general there is very poor clinical prognosis even after peripheral nerve injury in humans. Modest PNS repair can be attributed to factors including poor axonal growth due to the hostile injury-induced microenvironment, misrouting or re-innervation errors due to the gradual loss of axon guiding, regenerative signals [95]. These challenges further compound the difficulties stemming from the slow rate of axonal growth [96]. As a consequence, regenerating axons often fail to reach their original innervation targets, and functional restoration does not occur. Although full axonal regeneration is the ultimate goal for complete functional recovery, studies have demonstrated that promoting the remyelination of surviving PNS as well as CNS axons is sufficient to restore locomotion in previously paralysed mice [28,86,87]. As such, strategies to accelerate remyelination in order to prevent progressive axon degeneration have been recognized as a promising approach for spinal cord injuries [22]. Going forward, this review will focus on the use of SCs for central remyelination. For more details about SCs for CNS repair, we refer readers to these excellent reviews [89,97].

## 8. Why can we not simply transplant Schwann cells?

SCs for autologous transplantation can be isolated from peripheral nerve biopsies and are easily expandable in culture (reviewed in [98]), raising the question why one would not simply use patient-derived SCs? Despite the benefits of SC remyelination, the relapsing-remitting, multi-focal nature of demyelinating diseases, such as multiple sclerosis, limit the therapeutic feasibility of SC transplantation. In order to fill the many lesions, patients would require multiple invasive transplantation surgeries because the systemic delivery of SCs is likely to show limited success, due to the poor migratory abilities of SCs in the normal white matter. Although SCs are intrinsically a mobile, migratory cell type, the presence of reactive astrocytes greatly restricts its movement in culture [99] and in the CNS [4,6,72,100]. Therefore, unless the demyelinating injury also damages astrocytes, transplanted SCs will typically be prevented from migrating within and beyond the injection site, which significantly limits the extent of functional restoration [101]. Poor SC migration within the normal white matter impinges upon its survival and integration within the CNS, because SCs are less likely to make contact with axons and receive neuronal-derived growth factors [3]. Indeed, tracking of prelabelled SCs found few surviving SCs four weeks post-transplantation. The cells that did survive long term were cells in astrocyte-free zones that had made contact with axons [102,103]. Despite the promising repair capacity of SCs, they are poorly tolerated in the CNS after transplantation. Therefore, in addition to optimizing the routes of cell administration, modifications to the exogenous SCs or CNS environment need to be introduced to overcome the limitations of SC transplantation [104–106].

## 9. Direct differentiation of oligodendrocyte progenitor cells to Schwann cells can circumvent transplantation limitations

Unlike SCs, OPCs are widely distributed throughout the white and grey matter and can migrate towards and within the lesion zone during the recruitment phase (figure 3*c*) following demyelination. Furthermore, OPCs, a CNS progenitor cell, can interact with other CNS components without eliciting a reactive response. Thus, it may be possible to override SC inhibitory astrocytic signals by directly converting existing OPCs, which are already distributed throughout the CNS, into SCs by exogenously activating the alternative OPC cell fate programme. In this way, SCs are more likely to form in astrocyte rich areas, allowing greater SC presence within the CNS, and importantly also in regions that would not be accessible for transplantation. Importantly, direct differentiation of OPCs into SCs should better disperse SCs within the CNS and place them in closer proximity to demyelinated axons. This should increase survival rates since SCs are reliant upon axonally derived survival factors such as NRG1 [107]. Interestingly, once they associate with axons and are in receipt of growth factors, SCs are much more resistant to the inhibitory effects of astrocytes, allowing their stable integration within the CNS. Indeed, when SCs do survive in the CNS, their myelin sheaths are stably maintained for more than a year post-transplantation in lesioned rodents [84,108] and for over a decade after spinal cord injury in humans [10].

## 10. Potential considerations of using Schwann cell differentiation for central nervous system remyelination therapies

The caveats of encouraging widespread SC-mediated CNS remyelination lie in the potential consequences of substituting oligodendrocytes with SCs. While there are many striking similarities between the two myelinating cells, their separate evolutionary developments have also yielded functional nuances. Of particular concern is the potential increase in volume and metabolic demand, since SCs, unlike oligodendrocytes can only form one myelin sheath around an axon segment. Therefore, greater numbers of SCs may be required to replace lost oligodendrocytes, since each cell can only ensheath a single axon. However, whether sufficient numbers of SCs can be generated by the exogenous activation of OPCs to restore information transmission efficiency is presently unclear. Each individual SC possesses a large, non-axonal cytoplasmic and nuclear domain. Thus, compared to central white matter, the peripheral nerve shows lower compaction, and therefore reduced axon density and decreased information transmission per unit volume. Indeed, SC-remyelinated CNS axons similarly exhibit characteristic peripheral nerve features, including thicker myelin, enlarged extracellular space and deposition of extracellular collagen [19]. However, ultimately, the true extent of SC remyelination will likely be limited by the physical and metabolic constraints of the CNS. Taken together, SC myelination therapies may not only represent a novel therapy to circumvent the limitations of transplantation, but also provide new opportunities to challenge CNS plasticity.

Functional incongruity may also arise due to differences in axon selection and in myelination patterns. Within the CNS, all axons greater than 0.4 μm in diameter are myelinated by oligodendrocytes, whereas in the periphery, SCs only myelinate axons larger than 1 µm in diameter. Although myelinating small-diameter axons is typically avoided because it is more energetically costly [109], within regions such as the optic nerve, this expense can be justified to facilitate the rapid transduction and synchronization of signals needed to perceive and respond to the external environment. Therefore, it will be of functional importance to evaluate whether small-diameter axons, if demyelinated, could be impervious to SC remyelination therapy. Furthermore, oligodendrocytes intriguingly myelinate axonal segments discontinuously within the superficial layers of the cortex, resulting in a patchy myelination pattern [110]. By contrast, peripheral axons are considered uniformly ensheathed along its entire length, although intermittent myelination has been observed in *Xenopus* [111]. If SCs are unable to faithfully recapitulate these myelination patterns, adaptions to such differences could affect the precision and timing of action potentials within the entire neuronal network, leading to incomplete functional restoration, or even behavioural deficits. Whether the CNS, eventually learns to adapt to the consequences of peripheral-like reorganization requires further investigation.

Beyond electrophysiological function, whether SCs and oligodendrocytes are functionally interchangeable will be of utmost importance for the long-term survival of central axons. Although still poorly understood, observations from leukodystrophies have pointed towards axonal support as a critical function of myelin. In the CNS of terrestrial vertebrates, myelin proteolipid proteins (PLPs) represent the dominant protein family [112], whereas the PNS, as well as the CNS of aquatic vertebrates, predominantly express myelin protein zero (P0). Structurally, PLPs differ from the older DM20 isoform by the addition of 35 amino acids encoding a second intracellular loop [113,114]. This novel mutation is thought to increase compaction of PLP myelin, which allowed PLP to better adapt to the spatial constraints of the CNS. Animals that were engineered to express P0, instead of PLP in CNS myelin showed increased myelin degeneration, decreased motor function and shortened lifespan [115]. Thus, while P0 can substitute for PLP in the short run, CNS axons are likely to have evolved distinct metabolic requirements, that CNS myelin-associated proteins, including PLP are uniquely positioned to provide. Myelin that contained both PLP and P0, remained mostly indistinguishable from that of CNS myelin, raising the possibility that PNS myelin proteins can conform to CNS constraints when given sufficient context. Presently, few studies have been able to assess the long-term consequences of widespread CNS SC remyelination, because the number of SC remyelinated axons are typically low compared to the oligodendrocyte remyelinated majority. Thus, functional changes observed after SC remyelination may be confounded by compensatory mechanisms of oligodendrocytes [108]. However, it will be important to assess these potential consequences, because functional nuances arising from differences in myelin protein composition may become difficult to mask when SCs remyelination is enhanced. Moreover, SCs and oligodendrocytes have different metabolic needs and strategies [116], which might impact the ability of SCs in the CNS to provide the appropriate metabolites in a sufficient quantity to CNS axons. Therefore, for SCs to be a viable remyelination therapy, it will be important to ensure that peripheral myelin, even against the backdrop of central myelin, can provide sufficient trophic support.

## 11. Conclusion

The finding that OPCs are a key source of CNS-SCs opens up new endogenous targets to achieve regeneration following demyelinating diseases. Indeed, compelling evidence from transplantation studies have demonstrated that transplanted SCs can extensively promote remyelination and functional restoration in the demyelinated spinal cord. Axons myelinated by SCs may prove less vulnerable to immune attacks targeted against oligodendrocytes and their myelin sheaths [117]. Despite these functional benefits, SC therapies have been greatly hindered by logistical limitations. Instead, direct activation of the endogenous OPC to SC differentiation pathways represents new strategies to maximally implement SC for remyelination therapy. Many studies have contributed to the current understanding of the major mechanistic and regulatory elements of OPC to SC differentiation. However, many questions still remain. What are the molecular mechanisms driving the OPC to SC differentiation? What intrinsic or extrinsic cues can be targeted for the directed differentiation of OPCs into SCs? What is the functional state and molecular signatures of CNS-SCs, and how can it be modified to enhance CNS repair? How similar are CNS and PNS SCs? Can CNS-derived SCs replace all functions of oligodendrocytes in the CNS? Resolution of these questions will provide important insight into the successful development of SC-mediated therapies to restore cytoarchitecture and function to CNS axons after demyelination.

## Data accessibility

This article has no additional data.
